# Cloning, Identification and Functional Characterization of Bovine Free Fatty Acid Receptor-1 (FFAR1/GPR40) in Neutrophils

**DOI:** 10.1371/journal.pone.0119715

**Published:** 2015-03-19

**Authors:** Carolina Manosalva, Jaqueline Mena, Zahady Velasquez, Charlotte K. Colenso, Sebastian Brauchi, Rafael A. Burgos, Maria A. Hidalgo

**Affiliations:** 1 Laboratory of Molecular Pharmacology, Institute of Pharmacology, Faculty of Veterinary Science, Universidad Austral de Chile, Valdivia, Chile; 2 Department of Biology, Universidad de Nariño, Pasto, Colombia; 3 Institute of Physiology, Faculty of Medicine, Universidad Austral de Chile, Valdivia, Chile; CRCHUM-Montreal Diabetes Research Center, CANADA

## Abstract

Long chain fatty acids (LCFAs), which are ligands for the G-protein coupled receptor FFAR1 (GPR40), are increased in cow plasma after parturition, a period in which they are highly susceptible to infectious diseases. This study identified and analyzed the functional role of the FFAR1 receptor in bovine neutrophils, the first line of host defense against infectious agents. We cloned the putative FFAR1 receptor from bovine neutrophils and analyzed the sequence to construct a homology model. Our results revealed that the sequence of bovine FFAR1 shares 84% identity with human FFAR1 and 31% with human FFAR3/GPR41. Therefore, we constructed a homology model of bovine FFAR1 using human as the template. Expression of the bovine FFAR1 receptor in Chinese hamster ovary (CHO)-K1 cells increased the levels of intracellular calcium induced by the LCFAs, oleic acid (OA) and linoleic acid (LA); no increase in calcium mobilization was observed in the presence of the short chain fatty acid propionic acid. Additionally, the synthetic agonist GW9508 increased intracellular calcium in CHO-K1/bFFAR1 cells. OA and LA increased intracellular calcium in bovine neutrophils. Furthermore, GW1100 (antagonist of FFAR1) and U73122 (phospholipase C (PLC) inhibitor) reduced FFAR1 ligand-induced intracellular calcium in CHO-K1/bFFAR1 cells and neutrophils. Additionally, inhibition of FFAR1, PLC and PKC reduced the FFAR1 ligand-induced release of matrix metalloproteinase (MMP)-9 granules and reactive oxygen species (ROS) production. Thus, we identified the bovine FFAR1 receptor and demonstrate a functional role for this receptor in neutrophils activated with oleic or linoleic acid.

## Introduction

Neutrophils are the first line of host defense, and they are one of the first cells to migrate from the blood into injured or infected tissues. Neutrophils exert their defensive role through oxidative and non-oxidative mechanisms; oxidative mechanisms are associated with ROS production, such as superoxide, via activating the NADPH oxidase complex [1], whereas the non-oxidative mechanisms include the release of granules that contain proteolytic proteins, such as MMP-9 [2]. Granule release is triggered by several stimuli that, upon coupling to exposed extracellular receptors, produce signals across the plasma membrane, which increase the intracellular generation of second messengers. The process involved in the secretion of MMP-9 granules is only partially understood, but several authors have demonstrated that the increase in intracellular calcium is an important signal [3,4].

Fatty acids regulate immune and inflammatory responses in humans [5,6]. In the bovine, free fatty acids are significantly increased in the plasma between 1 to 2 weeks postpartum [7], the same period during which cows are more susceptible to acquire infectious diseases that the innate immune system actively opposes. Linoleic acid (C18:2), a polyunsaturated long chain fatty acid (LCFA), is the main fatty acid in plasma and its percentage significantly increases two weeks postpartum compared with values before parturition. Similarly, the unsaturated LCFA oleic acid (C18:1), also shows an increase until two weeks postpartum [7]. In vitro, fatty acids affect ROS production in neutrophils. Oleic acid, linoleic acid and γ-linolenic acid, markedly increase intracellular and extracellular ROS levels in rat and human neutrophils [8]. In bovine neutrophils, oleic acid increases intracellular superoxide levels in an intracellular calcium-dependent manner [4]. Oleic and linoleic acids also rapidly increase MMP-9 activity in bovine neutrophils in an extracellular calcium-dependent manner [4,9]. Similarly, oleic acid promotes MMP-9 secretion in breast cancer cells through PKC, Src and EGFR-dependent pathways [10]. Despite the fact that oleic and linoleic acids induce MMP-9 release in breast cancer cells and bovine neutrophils, the mechanisms underlying this response are not well understood.

Recent studies demonstrated that free fatty acids are ligands for seven-transmembrane domain receptors. Free Fatty Acid Receptor 1 (FFAR1), also known as G Protein-coupled Receptor 40 (GPR40), is a receptor for medium and long chain fatty acids, such as docosahexaenoic acid (DHA), eicosapentaenoic acid (EPA), oleic acid and linoleic acid [9,11,12,13]. FFAR1 mediates insulin secretion from pancreatic β-cells; however, more recent evidence suggests that FFAR1 plays a role in cellular proliferation and innate immunity because it is present in human and bovine mammary epithelial cells [14,15] and bovine neutrophils [4]. In pancreatic β-cells and breast cancer cells, FFAR1 is coupled to an intracellular G_q_ protein that activates the PLC and phosphatidylinositol-4,5-bisphosphate signaling pathways [16,17]. FFAR1 is also coupled to G_i_ protein in bovine neutrophils because pertussis toxin only partially reduces oleic acid-induced intracellular calcium mobilization [4]. Several effects of oleic and linoleic acid have been described in bovine neutrophils [4,9], and treatment with the synthetic FFAR1 antagonist, GW1100, suggested that FFAR1 plays a role in neutrophil activation [9].

Recently, several discrepancies regarding the identity of the FFAR1 cDNA sequence in bovine have arose. The first antecedents of FFAR1 showed a cDNA sequence obtained from bovine mammary epithelial cells with 84% identity to human FFAR1 [15]. In bovine neutrophils, we observed a product of FFAR1 amplification that was similar to that observed in bovine mammary epithelial cells using RT-PCR. Additionally, a protein of 31 kDa was detected using an antibody against human FFAR1, which is similar to the size of human FFAR1; this protein was also visualized using confocal microscopy [4]. However, a modification in the identity of this sequence was recently introduced in GenBank (GenBank Accession No. **XM_870502.1**). Due to standard genome annotation processing predicted by automated computational analysis, this sequence was identified as part of a transcription variant of the short chain fatty acid receptor, FFAR3 (GPR41) (GenBank Accession No. **XR_238380.1**). Although computational analysis named this sequence as a transcription variant of FFAR3 due to its similarity with FFAR3, analysis and functional studies have not been performed to demonstrate whether this sequence is a receptor for short chain fatty acids or long chain fatty acids.

In the present study, we cloned the putative bovine cDNA FFAR1 receptor obtained from bovine neutrophils and analyzed its similarity to human FFAR1. Then, we evaluated the effects of activating the bFFAR1 receptor using synthetic or natural ligands of long and short chain fatty acid receptors in both CHO-K1 cells expressing the bFFAR1 receptor and bovine neutrophils. Finally, we examined the intracellular mechanisms involved in MMP-9 release and ROS production after FFAR1 receptor activation.

## Materials and Methods

### Neutrophil isolation

**Fig 1:**
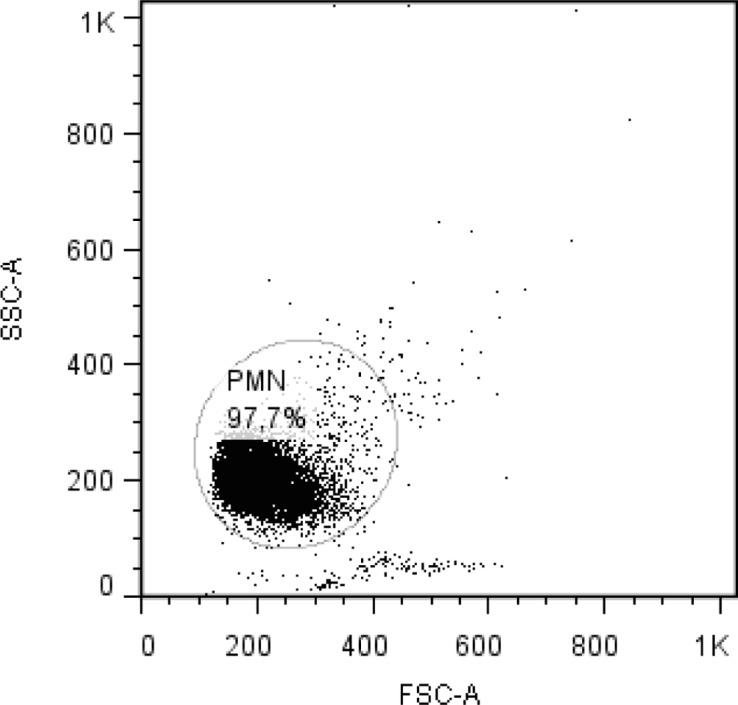
Analysis of neutrophil purity by flow cytometry. A forward-scatter vs side-scatter dot plot from a sample of neutrophils (PMN) after isolation procedure is shown.

Blood was collected by jugular venipuncture of five healthy Holstein heifers from a Universidad Austral de Chile herd, and samples were collected in ACD Blood Collection Tubes (Becton Dickinson, USA). All experiments were conducted in strict accordance with protocols approved by the Ethical Committee of the Universidad Austral de Chile (Permit Number: 06/10). Neutrophils were isolated according to a previously described method [18]. Viability was determined by trypan blue exclusion assays, and it was at least 97% for all experiments. Neutrophil purity was at least 94% (Fig. 1), as assessed by flow cytometry (BD FACSCanto II, USA) using a forward-scatter versus side-scatter dot plot to determine the relative size and granularity of cells [19].

### Cloning of bovine FFAR1 receptor

Total RNA was extracted from bovine neutrophils using the Total RNA Kit I (Omega Bio-Tek Inc, Norcross, GA, USA), according to the manufacturer's instructions. RNA was treated with 2 U/μL DNase I (Life Technologies, Carlsbad, CA, USA) to remove traces of genomic DNA. For cDNA synthesis, 250 ng of total RNA was used with reverse transcriptase (M-MLV Reverse Transcriptase, Promega, Madison, WI, USA), according to the manufacturer's instructions. Putative bovine FFAR1 (bFFAR1) receptor cDNA was obtained by PCR amplification using the following oligonucleotides synthesized based on the genomic DNA sequence of the putative bFFAR1 receptor (GenBank Accession No. **XM_870502**): 5′- CTCGAGGGTACCATGGACCTGCCCCCGCAGCTC—3′ (forward) and 5′- GCGGCCGCGGATCCCTATTTCTGGGATGCCCCTGC—3′ (reverse). PCR was performed using Green Master Mix DNA polymerase and Pfu DNA Polymerase (1:3) under the following conditions: 1 min at 95°C; 35 cycles of 20 s at 95°C, 20 s at 60°C and 1 min at 72°C; and 3 min at 72°C. A fraction of the PCR product was resolved on a 1.2% agarose gel, and the band was excised from the gel, purified and subcloned into the pGEM T-Easy vector (Promega, Madison, WI, USA). Plasmids from the resulting bacterial clones were isolated, and the full insert was verified by sequencing. Next, pcDNA3.1 (Invitrogen, Carlsbad, CA, USA) was digested with NotI and BamHI (New England Biolabs Inc., Ipswich, MA, USA) and bFFAR1 cDNA was ligated at both ends. bFFAR1 in pcDNA3.1 (pcDNA3.1-bFFAR1) was successfully transfected into CHO-K1 cells using FuGene 6 reagent (Promega, Madison, WI, USA), according to the manufacturer's instructions.

### Sequence analysis and homology modeling

**Fig 2:**
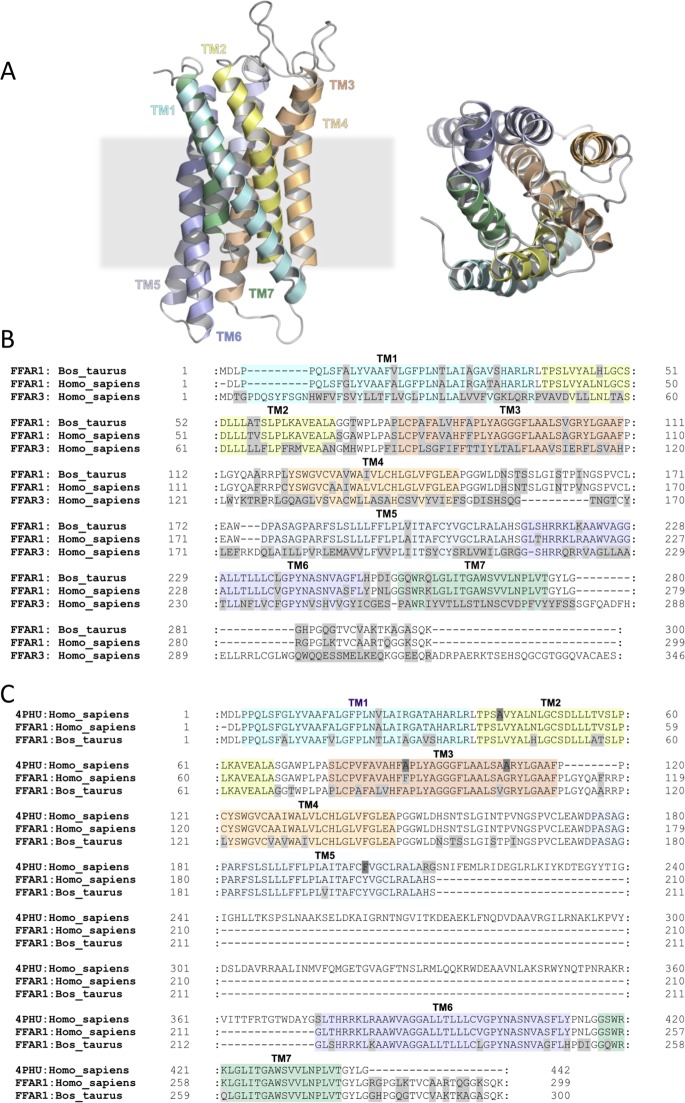
Sequence analysis and homology modeling of the bovine FFAR1 receptor. Bovine FFAR1 receptor cDNA was amplified from total RNA obtained from bovine neutrophils by RT-PCR, cloned, sequenced, and the predicted amino acid sequence was obtained. (A) Structure of the bovine FFAR1 membrane domain homology model; side-view (left) and top-view (right). (B) Sequence alignment between the putative bovine FFAR1 membrane domain and the human FFAR1 (4PHU: Homo_sapiens (PDB: 4PHU) and FFAR1: Homo_sapiens (accession number: AAH95536)) used for model construction. Colored sequence annotations based on 4PHU identify the extent of transmembrane helices. Residues highlighted in grey indicate a mismatch between the aligned sequences. 4PHU residues highlighted in dark grey indicate point mutations made to aid structure determination (Leu^42^Ala, Phe^88^Ala, Gly^103^Ala and Tyr^202^Phe). (C) Sequence alignment between the putative bovine FFAR1 membrane domain, human FFAR1 and human FFAR3 was performed using Clustal Omega. Colored sequence annotations based on 4PHU identify the approximate extent of transmembrane helices. Residues highlighted in grey indicate a mismatch between the aligned sequences.

The bFFAR1 cDNA and predicted protein sequence were analyzed using the BLAST search [20] and Clustal Omega [21]. The predicted protein sequence was obtained using EMBOSS Transeq (http://www.ebi.ac.uk/Tools/st/emboss_transeq/). A non-hydrogen atom homology model of bovine FFAR1 was obtained using the automated methodology implemented in MODELLER [22] (Fig. 2A). The crystal structure of human FFAR1 was used as template. A sequence alignment produced from Clustal Omega and refined manually was used as the input for model construction (Fig. 2B). The C-terminal bovine sequence, G280-K300, was omitted from the model due to the absence of these residues in the crystal structure. The stereochemical quality of the bovine FFAR1 homology model was evaluated using WHAT_CHECK [23].

### Transient transfection

Chinese hamster ovary (CHO)-K1 cells (ATCC CCL-61, Manassas, VA) were used to express the bFFAR1 protein and determine whether long or short chain fatty acids (oleic acid, linoleic acid, and propionic acid, respectively) or the synthetic agonist of FFAR1 (GW9508) activated this protein. CHO-K1 cells were chosen because they provide stable and accurate posttranslational modification, contain components for typical GPR signaling, and have no endogenous FFAR1 or FFAR3 expression [24,25,26]. CHO-K1 cells were maintained in Dulbecco's modified Eagle's medium/nutrient mixture F-12 (Invitrogen, Carlsbad, CA, USA) containing 10% fetal bovine serum (v/v) and 2 mM glutamine, at 37°C with 5% CO_2_. At approximately 50% confluence, the cells were transfected with pcDNA3.1-bFFAR1 (CHO-K1/bFFAR1 cells) or empty pcDNA3.1 plasmid (CHO-K1/pcDNA3.1 cells) using FuGene 6 as a transfection reagent (Roche, Indianapolis, IN). Forty-eight hours after transfection, the cells were subsequently treated with trypsin and suspended in HBSS for intracellular calcium mobilization or flow cytometry experiments.

### Flow cytometry

Confluent CHO-K1/bFFAR1 cells, CHO-K1/pcDNA3.1 cells or MCF-7 cells were trypsinized and collected by centrifugation. Five hundred thousand bovine neutrophils were centrifuged at 300 x g for 6 min. All cells were fixed for 10 min with 1% paraformaldehyde in PBS. Then, the cells were incubated overnight at 4°C in 1% BSA-PBST (PBS-Tween 0.1%) containing 1:100 rabbit anti-human FFAR1 (Cat. No. Ab75197, Abcam, Boston, MA, USA). A sample lacking the primary antibody was included as a control. Then, the cells were washed twice with PBS and incubated with 1% BSA-PBST with 1:1,000 goat anti-rabbit Alexa 488 (Life Technologies, Eugene, OR, USA), and incubated for 1 h at room temperature in the dark. After the cells were washed with PBS and suspended in 300 μl of PBS, they were analyzed by flow cytometry using FlowJo 7.6 software.

### Measurement of intracellular calcium levels in CHO-K1/bFFAR1 cells

CHO-K1/bFFAR1 or CHO-K1/pcDNA3.1 cells (2 × 10^6^ cells/2 ml) were loaded with 2.5 μM Fura-2AM fluorescent indicator dye (Sigma-Aldrich, Saint Louis, MO, USA) in recording buffer (10 mM HEPES, 140 mM NaCl, 2 mM CaCl_2_, 21 mM MgCl_2_, 25 mM KCl, 10 mM glucose, pH 7.4) for 30 min, washed three times with recording buffer, and returned to the incubator for 10 min. Cells were incubated with different concentrations of propionic acid (1, 10 and 30 mM) (Sigma-Aldrich, Saint Louis, MO, USA), oleic acid (0–500 μM) (Sigma-Aldrich, Saint Louis, MO, USA), linoleic acid (0–200 μM) (Sigma-Aldrich, Saint Louis, MO, USA), GW9508 (0–100 μM) (Cayman Chemical, Ann Arbor, MI, USA), ionomycin (2 μM) (Sigma-Aldrich, Saint Louis, MO, USA), thapsigargin (2 μM) (Sigma-Aldrich, Saint Louis, MO, USA) or vehicle (0.1% DMSO). The fatty acid concentrations used in all experiments are in the range of concentrations of healthy and peripartum cows [27,28]. In another set of experiments, cells were incubated with either 10 μM GW1100 (Cayman Chemical, Ann Arbor, MI, USA) for 15 min, 2 μM U73122 (Tocris, Bristol, United Kingdom) for 3 min or vehicle (0.1% DMSO) for 15 min and then stimulated with either 300 μM oleic acid, 100 μM linoleic acid or 10 μM GW9508. Cellular fluorescence (Ca^2+^) was measured at 509 nm emission with 340/380 nm dual wavelength excitation using a LS55 spectrofluorimeter (PerkinElmer Life Science). Cuvette temperatures were maintained at 37°C with constant stirring.

### Measurement of intracellular calcium levels in bovine neutrophils

Neutrophils (1 × 10^7^ cells/ml) were suspended in HBSS and incubated with 2 μM Fluo-4 AM (Molecular Probes, Eugene, OR, USA) for 30 min at 37°C. The cells were washed 3 times, and 1×10^6^ Fluo-4 AM-loaded cells suspended in 250 μl HBSS supplemented with 0.9 mM CaCl_2_ were incubated for 5 min at 37°C in a microplate. Neutrophils were incubated with vehicle (0.1% DMSO) or either 10 μM GW1100 or 2 μM U73122 for 15 or 3 min, respectively. A basal fluorescent signal was measured, followed by the addition of 300 μM oleic acid or 100 μM linoleic acid. As control, ionomycin (2 μM) and thapsigargin (2 μM) were used. Cellular fluorescence (Ca^2+^) was measured at 526 nm with excitation at 488 nm using a Varioskan microplate reader (Thermo Scientific, Rockford, IL, USA).

### Determination of MMP-9 activity

One million neutrophils in 500 μl of HBSS were incubated with vehicle or either 10 μM GW1100, 2 μM U73122 or 1 μM staurosporine (Selleckchem, Houston, TX, USA) for 15, 3 or 15 min, respectively, at 37°C. Then, neutrophils were stimulated with 300 μM oleic acid, 100 μM linoleic acid, 10 μM GW9508 or vehicle for 5 min at 37°C. As positive control, 100 nM platelet activating factor (PAF) for 5 min at 37°C was used. After incubation, the cells were centrifuged at 600 × g for 6 min, and equal amount of supernatants were assayed for gelatinase activity by zymography [29,30]. Ten microliters of supernatant was loaded into 10% polyacrylamide gels (0.75 mm thick) containing 0.28% gelatin. The gels were run at 200 V for 1 h in a Bio-Rad Mini Protean II apparatus (Bio-Rad Laboratories, Richmond, CA) and soaked twice in 2.5% Triton X-100 in distilled water on a shaker at room temperature for 30 min. Then, the gels were soaked in reaction buffer consisting of 100 mM Tris (pH 7.5) and 10 mM CaCl_2_ at 37°C overnight. The gels were stained in 0.5% Coomassie Brilliant Blue R-250 in acetic acid:methanol:water (1:3:6). Enzymatic activity was determined by measuring non-staining areas where the gelatin was degraded. Gelatinolytic bands were compared with a recombinant MMP-9 standard (Sigma-Aldrich, Saint Louis, MO, USA). To measure activity, the gels were digitalized, and the band intensity was determined using ImageJ 1.35s software.

### ROS production

Neutrophils (1 × 10^6^ cells) were loaded with 10 μM hydroethidine (HE) (Molecular Probes, Eugene, OR, USA) for 5 min at 37°C. Then, 2.5 × 10^5^ cells were incubated with vehicle, 10 μM GW1100 for 15 min, 2 μM U73122 for 3 min, 1 μM staurosporine for 15 min or 10 μM diphenyleneiodonium (DPI) (Sigma-Aldrich, Saint Louis, MO, USA) for 30 min at 37°C. Then, neutrophils were treated with vehicle, 300 μM oleic acid, 100 μM linoleic acid or 100 μM GW9508, for 5 min at 37°C. Fluorescence was analyzed using a flow cytometer and FlowJo 7.6 software (Tree Star, Inc. USA).

### Statistical analysis

All experimental protocols were performed in quintuplicate. Experiments in neutrophils were done from 5 different animals. Bar graphs present the arithmetic mean ± SEM. For statistical analyses, a one-way analysis of variance (ANOVA) and Dunnett’s multiple comparison test, and t-tests were performed. All analyses were performed using Graph Pad Prism v5.0 software, assuming that a p-value less than 0.05 represents a significant result.

## Results

### Sequence analysis of the bovine FFAR1 receptor

To analyze the sequence of the bovine FFAR1 receptor, we cloned the bovine FFAR1 from mRNA obtained from bovine neutrophils into the pGEM T-Easy vector and sequenced the obtained 902 bp fragment. A nucleotide BLAST search of the cDNA sequence showed 100% identity to a predicted Bos taurus FFAR3 transcription variant X2 [GenBank Accession No. **XR_238380.1**], 84% identity to human FFAR1 [GenBank Accession No. **EU432113.1**] and no significant similarity to human FFAR3 [GenBank Accession No. **EU432115.1**]. Next, we obtained the predicted protein sequence (300 amino acids) of the bovine cDNA FFAR1 receptor and performed a protein BLAST search [20] of putative bovine FFAR1. This search indicated that the bovine FFAR1 sequence shares more similarity with human FFAR1 compared to human FFAR3. Furthermore, sequence alignment performed using Clustal Omega [21] revealed that bovine FFAR1 shares 84% identity with human FFAR1 (GenBank Accession No. **AAH95536.1**) and only 31% identity with human FFAR3 (GenBank Accession No. **NP_005295.1**) (Fig. 2C). Using the predicted protein sequence of the bovine FFAR1 receptor, we constructed a homology model of bovine FFAR1 using the recently published human FFAR1 crystal structure (PDB: 4PHU) [31] at 2.3 Å resolution as template. In summary, sequence analysis and homology modeling of the bovine FFAR1 receptor strongly suggests that its sequence is more related to the FFAR1 receptor than the FFAR3 receptor.

### LCFAs increase intracellular calcium in CHO-K1/bFFAR1 cells

**Fig 3:**
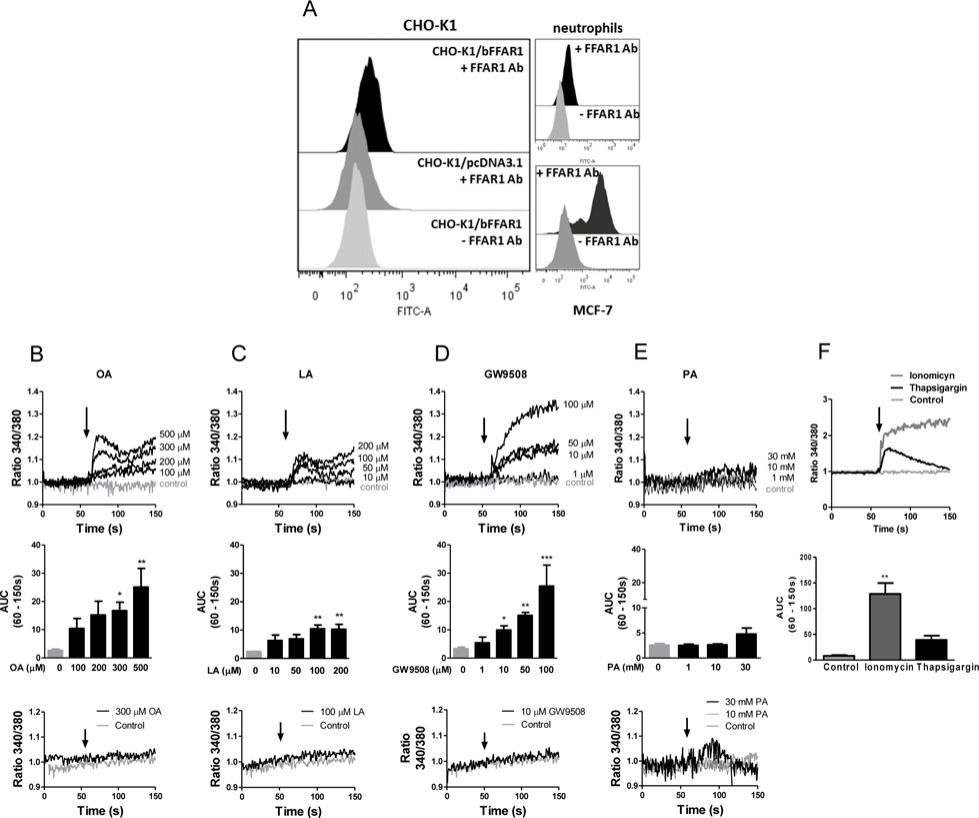
LCFAs and GW9508 increase intracellular calcium mobilization in CHO-K1 cells expressing bovine FFAR1 receptor. CHO-K1 cells were transfected with pcDNA3-bFFAR1 (CHO-K1/bFFAR1) or empty pcDNA3.1 plasmid (CHO-K1/pcDNA3.1), and the expression of bFFAR1 receptor was analyzed by FACS (A). Bovine neutrophils and MCF7 cells were used as positive controls. + FFAR1 Ab: analysis with FFAR1 antibody (primary antibody);—FFAR1: analysis without FFAR1 antibody (primary antibody). Intracellular calcium in CHO-K1/bFFAR1 cells was measured by spectrofluorimetry using the fluorescent probe Fura2-AM (B-F). A representative register (upper graphs) and area under the curve (AUC) between 60–150 s (bar graphs in middle graph) of different concentrations of oleic acid (0–500 μM) (B), linoleic acid (0–200 μM) (C), GW9508 (0–100 μM) (D), propionic acid (0–30 mM) (E) or ionomycin (2 μM) and thapsigargin (2 μM) (F) are shown. In each set of experiments, a representative register indicates treatment with solvent (control), 300 μM oleic acid, 100 μM linoleic acid, 10 μM GW9508 or 10 and 30 mM propionic acid in CHO-K1 cells transfected with empty pcDNA3.1 plasmid (bottom graphs). Arrows represent the time of solvent (control) or ligand addition. Mean ± SEM of 5 independent experiments. * p < 0.05, ** p < 0.01, *** p < 0.001 compared with the control. OA: oleic acid, LA: linoleic acid, PA: propionic acid.

The human FFAR1 receptor (hGPR40) is activated by long and medium chain free fatty acids, and its activation increases intracellular calcium in CHO cells overexpressing this receptor (CHO-hGPR40) [25]. We cloned the putative bovine FFAR1 cDNA into the pcDNA3.1 expression plasmid and transiently transfected CHO-K1 cells with pcDNA3.1-FFAR1 (CHO-K1/bFFAR1 cells) or empty pcDNA3.1 vector (CHO-K1/pcDNA3.1 cells). Next, we used FACS to evaluate the expression of bFFAR1 and the ability of the transfected cells to increase intracellular calcium levels after stimulation with different concentrations of propionic acid, oleic acid, linoleic acid or the synthetic FFAR1 agonist, GW9508. FACS analysis revealed that fluorescence increased in CHO-K1/bFFAR1 cells, but not in CHO-K1 cells transfected with the empty vector, indicating that CHO-K1/bFFAR1 cells expressed the bFFAR1 receptor (Fig. 3A). Bovine neutrophils and MCF-7 cells served as controls because these cells express the FFAR1 receptor [4,14]. As a negative control, all cell types were incubated without the FFAR1 antibody, producing a lower signal compared with cells incubated with the primary antibody.

Next, we analyzed the intracellular calcium mobilization in Fura 2-AM-loaded CHO-K1/bFFAR1. We observed that different concentrations of the LCFA, oleic (100–500 μM) and linoleic (50–200 μM), and the synthetic FFAR1 receptor agonist, GW9508 (10–100 μM), increased intracellular calcium (Fig. 3 B-D, upper graphs). The curve representing the oleic and linoleic acid-induced increase in intracellular calcium was biphasic, displaying a rapid increase, followed by a slight decrease, and a second phase of increase. In contrast, the GW9508-induced intracellular calcium response displayed a rapid and sustained increase. The analysis of the area under the curve (AUC) between 60–150 s (AUC_(60–150 s)_) revealed a significant increase in intracellular calcium induced by oleic acid at 300 and 500 μM, linoleic acid at 100 and 200 μM and GW9508 at 10, 50 and 100 μM (Fig. 3B-D, middle graphs). Incubation of CHO-K1/bFFAR1 cells with different concentrations of propionic acid, a short chain fatty acid, did not increase intracellular calcium (Fig. 3E). Ionomycin (2 μM) and thapsigargin (2 μM), positive controls, increased the intracellular calcium in CHO-K1/bFFAR1 cells, being the response stimulated by ionomycin higher compared with thapsigargin (Fig. 3F). Additionally, intracellular calcium levels did not increase in cells transfected with empty pcDNA3.1 vector after stimulation with each stimulus (Fig. 3B-E, bottom graphs). Thus, these results indicate that intracellular calcium is only induced by LCFAs and the synthetic agonist of the FFAR1 receptor, but not by a short chain fatty acid, suggesting that the sequence cloned corresponds to a LCFA receptor.

### bFFAR1 activation increases intracellular calcium through PLC in CHO-K1/bFFAR1 cells

The human FFAR1 receptor is coupled to the Gαq protein, and it stimulates PLC activity [25,32]. To confirm the participation of the bFFAR1 receptor and investigate the mechanism by which it increases intracellular calcium in response to ligands of bFFAR1, we used the synthetic antagonist of the FFAR1 receptor, GW1100, and the PLC inhibitor, U73122.

**Fig 4:**
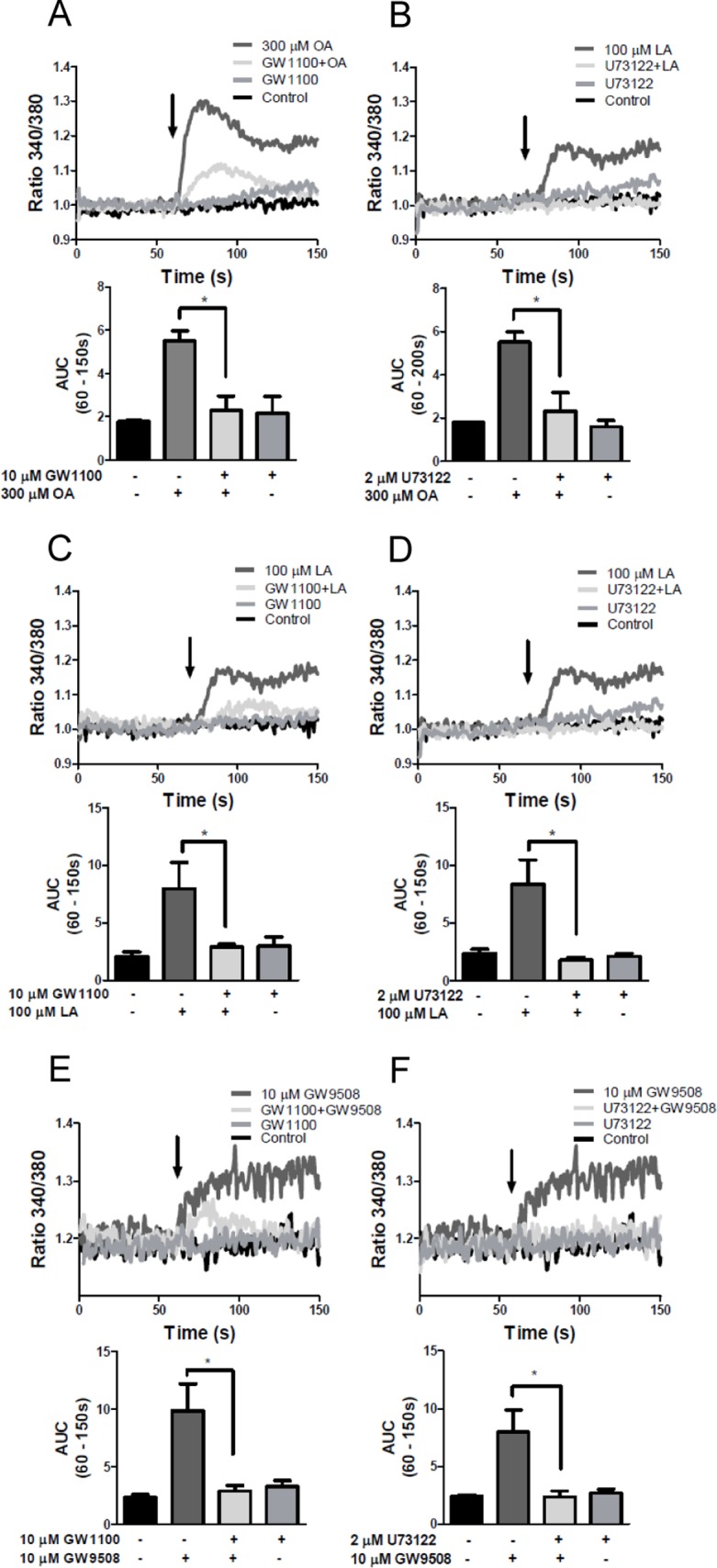
Pharmacological inhibition of the intracellular calcium mobilization induced by LCFAs or GW9508 in CHO-K1/bFFAR1 cells. CHO-K1 cells transfected with the bovine FFAR1 receptor were treated with solvent, the antagonist of the FFAR1 receptor, GW1100 (10 μM), for 15 min or the PLC inhibitor, U73122 (2 μM), for 3 min at 37°C, followed by solvent or 300 μM oleic acid (A and B), 100 μM linoleic acid (C and D) or 10 μM GW9508 (E and F). A representative experiment (upper graph) and area under the curve (AUC) between 60–150 s (bar graphs in lower graph) are shown. Arrows indicate the time of solvent or ligand addition. Mean ± SEM of 5 independent experiments. * p < 0.05. OA: oleic acid, LA: linoleic acid.

CHO-K1/bFFAR1 cells were incubated for 15 min with 10 μM GW1100 or vehicle (0.1% DMSO) and then stimulated with vehicle, oleic acid, linoleic acid or GW9508. GW1100 significantly reduced the increase in intracellular calcium induced by 300 μM oleic acid (AUC _(60–150 s)_, p < 0. 05, Fig. 4A), 100 μM linoleic acid (AUC _(60–150 s)_, p < 0. 05, Fig. 4C) and 10 μM GW9508 (AUC _(60–150 s)_, p < 0. 05 Fig. 4E).

To inhibit PLC activity, CHO-K1/bFFAR1 cells were incubated with 2 μM U73122 for 3 min and then stimulated with each stimulus. U73122 significantly decreased the induction of intracellular calcium in response to 300 μM oleic acid (AUC _(60–150 s)_ (p < 0. 05 Fig. 4B), 100 μM linoleic acid (AUC _(60–150s)_ (p< 0.05, Fig. 4D) and 10 μM GW9508 (AUC _(60–150 s)_ (Fig. 4F)). These results demonstrate that bGPR40 activation increases intracellular calcium via PLC activation.

### Oleic and linoleic acid increase intracellular calcium through bFFAR1 and PLC in bovine neutrophils

**Fig 5:**
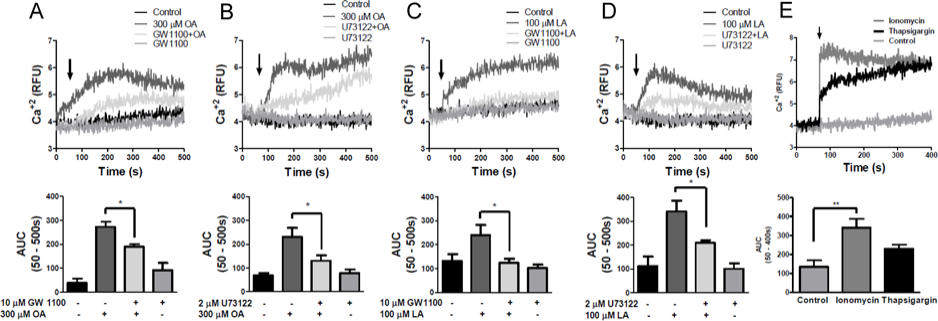
Pharmacological inhibition of the intracellular calcium mobilization induced by LCFAs in bovine neutrophils. Fluo4-AM-loaded neutrophils were treated with solvent, 10 μM GW1100 for 15 min or 2 μM U73122 for 3 min at 37°C, followed by solvent, 300 μM oleic acid (A and B) or 100 μM linoleic acid (C and D). As control, 2 μM ionomycin and 2 μM thapsigargin was used (E). A representative experiment (upper graph) and the area the under curve (AUC) between 60–150 s (bar graphs in lower graph) are shown. Arrows indicate the time of addition of the solvent or each fatty acid. Mean ± SEM of 5 independent experiments. * p < 0.05. OA: oleic acid, LA: linoleic acid.

Hidalgo *et al*., [4] demonstrated that bovine neutrophils express the FFAR1 receptor at the protein and mRNA levels, and they demonstrated that intracellular calcium increased in response to oleic and linoleic acid [4,9]. To demonstrate that oleic and linoleic acid increase intracellular calcium through FFAR1 activation in bovine neutrophils, we used the FFAR1 antagonist GW1100 to assess the role of FFAR1 in neutrophils. Once isolated from blood, these cells have a short life, making it difficult to silence gene expression. Fluo-4AM-loaded neutrophils were incubated with 10 μM GW1100 for 15 min, followed by the addition of ligand. GW1100 decreased the increase in intracellular calcium induced by 300 μM of oleic acid (AUC _(60–150 s)_ (p < 0.05, Fig. 5A) and 100 μM linoleic acid (AUC _(60–150 s)_ (p < 0.05, Fig. 5C). To assess whether the increase in intracellular calcium induced by LCFAs is mediated by PLC activation in bovine neutrophils, similar to CHO-K1/bFFAR1 cells, we incubated neutrophils with 2 μM U73122 for 3 min, followed by vehicle, oleic or linoleic acid. U73122 significantly decreased the increase in intracellular calcium levels induced by 300 μM oleic acid (AUC _(60–150 s)_ (p < 0.05, Fig. 5B) and 100 μM linoleic acid (AUC _(60–150 s)_ (p < 0.05, Fig. 5D). As controls, ionomycin and thapsigargin increased the intracellular calcium in bovine neutrophils (Fig. 5E). Thus, the increase in intracellular calcium levels in response to oleic or linoleic acid in bovine neutrophils is mediated by FFAR1 and PLC activation.

### bFFAR1 activation induces MMP-9 release via PLC and PKC in bovine neutrophils

**Fig 6:**
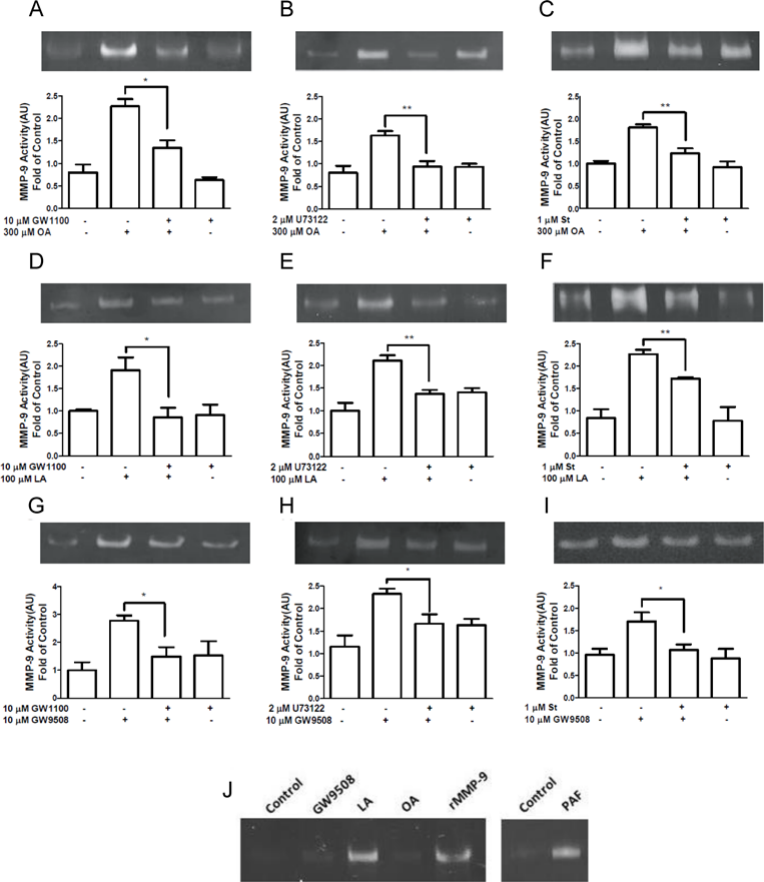
Effect of GW1100, U73122 or staurosporine on MMP-9 release induced by FFAR1 ligands in neutrophils. Bovine neutrophils were incubated with 10 μM GW1100 for 15 min, 2 μM U73122 for 3 min or 2 μM staurosporine for 15 min, prior to the addition of 300 μM oleic acid (OA) (A, B and C), 100 μM linoleic acid (LA) (D, E and F) or 10 μM GW9508 (G, H and I) for 5 min at 37°C. PAF (100 nM) as positive control was used, and recombinant MMP-9 (rMMP-9) as size control was loaded in the gel (J). Supernatants were collected and analyzed by zymography. Bar graphs represent the mean ± SEM of 5 independent experiments. AU: arbitrary units. * p < 0.05, ** p < 0.01. OA: oleic acid, LA: linoleic acid.

Intracellular calcium induces the release of cytoplasmic granules to extracellular medium [29]. Thus, because oleic and linoleic acid increase intracellular calcium and MMP-9 release in bovine neutrophils [4,9], we evaluated whether the FFAR1 receptor participates in MMP-9 granule release. We incubated bovine neutrophils with 10 μM GW1100 for 15 min, followed by vehicle, 300 μM oleic acid, 100 μM linoleic acid or 10 μM GW9508 for 5 min. Zymography assays revealed that 10 μM GW1100 significantly decreased MMP-9 activity in the supernatants of bovine neutrophils stimulated with 300 μM oleic acid (p < 0.05, Fig. 6A). Additionally, GW1100 decreased MMP-9 activity in bovine neutrophils stimulated with 100 μM linoleic acid (p < 0.05, Fig. 6D) or 10 μM GW9508 (p < 0.05, Fig. 6G). To evaluate the participation of PLC in MMP-9 release, bovine neutrophils were incubated with 2 μM U73122 for 3 min and then stimulated with each ligand for 5 min. U73122 significantly decreased MMP-9 activity in bovine neutrophils stimulated with 300 μM oleic acid (p < 0.01, Fig. 6B) and 100 μM linoleic acid (p < 0.01, Fig. 6E). Similarly, MMP-9 activity was decreased in neutrophils incubated with U73122 and then stimulated with 10 μM GW9508 (p < 0.05, Fig. 6H).

PLC activation triggers IP3 generation, the release of calcium from intracellular stores and activates PKC [33]. Moreover, PKC regulates IL-8-induced MMP-9 from human neutrophils [34]. To evaluate whether PKC participates in the FFAR1-ligand-induced MMP-9 release, we used an inhibitor of PKC, staurosporine. Bovine neutrophils were incubated with 1 μM staurosporine for 15 min, followed by vehicle, oleic acid, linoleic acid or GW9508 for 5 min. Staurosporine decreased MMP-9 release from bovine neutrophils stimulated with 300 μM oleic acid (p < 0.01, Fig. 6C) or 100 μM linoleic acid (p < 0.01, Fig. 6F). Additionally, MMP-9 release was significantly reduced in neutrophils treated with staurosporine and stimulated with 10 μM GW9508 (Fig. 6I). The controls PAF (positive control) and recombinant MMP-9 (size control) are shown in Fig. 6J.

Taken together, these results demonstrate that the PLC and PKC pathways mediate the FFAR1-ligand-induced release of MMP-9 granules.

### bFFAR1 ligands induce ROS production via FFAR1, PLC and PKC in bovine neutrophils

**Fig 7:**
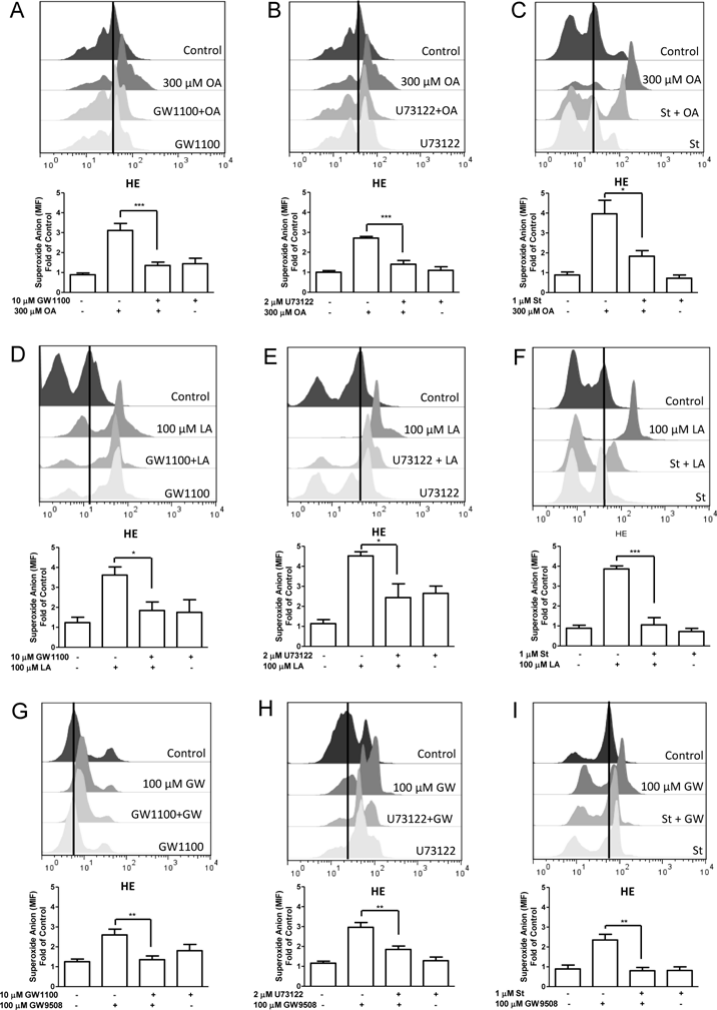
Effect of GW1100, U73122 or staurosporine on ROS production induced by FFAR1 ligands in neutrophils. Hydroethidine (HE)-loaded bovine neutrophils were incubated with 10 μM GW1100 for 15 min, 2 μM U73122 for 3 min or 2 μM staurosporine for 15 min, followed by the addition of 300 μM oleic acid (OA) (A, B and C), 100 μM linoleic acid (LA) (D, E and F) or 10 μM GW9508 (G, H and I) for 5 min at 37°C. The mean intensity fluorescence (MIF) was analyzed by FACS. Bar graphs represent the mean ± SEM of 5 independent experiments. * p < 0.05, ** p < 0.01, *** p < 0.001. OA: oleic acid, LA: linoleic acid, GW: GW9508.

Increases in intracellular calcium induce PKC activation and ROS production in human neutrophils [35]. Previously, we demonstrated that calcium participates in oleic acid-induced ROS production in bovine neutrophils [4]. Furthermore, polyunsaturated fatty acids can activate PKC in the rat brain [36]. To determine whether FFAR1 agonists induce ROS production via the activation of FFAR1 in bovine neutrophils, we incubated neutrophils with 10 μM GW1100 for 15 min, followed by vehicle, oleic acid, linoleic acid or GW9508. GW1100 significantly decreased ROS production in bovine neutrophils stimulated with 300 μM oleic acid (p < 0.001, Fig. 7A), 100 μM linoleic acid (p < 0.05, Fig. 7D) and 10 μM GW9508 (p < 0.01, Fig. 7G). To assess whether the increase in ROS production in response to oleic acid, linoleic acid and GW9508 is mediated by PLC, bovine neutrophils were incubated with 2 μM U73122 for 3 min, followed by each stimulus. In the presence of U73122, ROS production decreased in neutrophils stimulated with 300 μM oleic acid (p < 0.001, Fig. 7B), 100 μM linoleic acid (p < 0.05, Fig. 7E) and 10 μM GW9508 (p < 0.01, Fig. 7H). Then, we assessed the participation of PKC in FFAR1-agonist-mediated ROS production in bovine neutrophils incubated with 1 μM staurosporine for 15 min, followed by FFAR1 agonists. Our results demonstrate that staurosporine decreased the increase in ROS production stimulated by 300 μM oleic acid (p < 0.05, Fig. 7C), 100 μM linoleic acid (p < 0.001, Fig. 7F) and 100 μM GW9508 (p < 0.01, Fig. 7I). These results indicate that the increase in ROS production stimulated by oleic or linoleic acid, or GW9508 is mediated by FFAR1-PLC-PKC signaling in bovine neutrophils.

**Fig 8:**
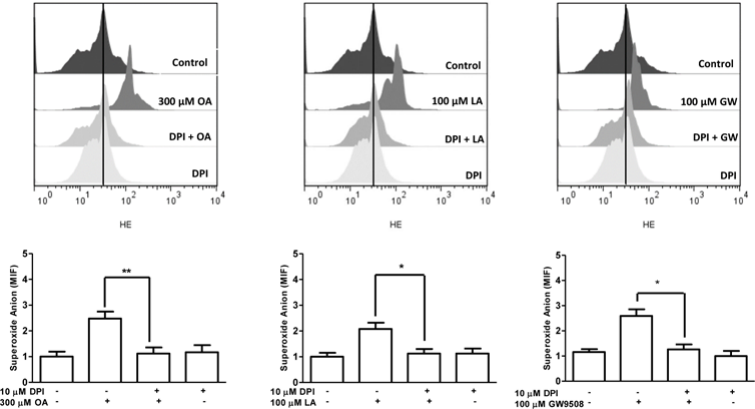
Diphenyleneiodonium (DPI) inhibits ROS production induced by FFAR1 ligands in neutrophils. Hydroethidine (HE)-loaded bovine neutrophils were incubated with 10 μM DPI for 30 min, followed by the addition of 300 μM oleic acid (OA), 100 μM linoleic acid (LA) or 10 μM GW9508 for 5 min at 37°C. The mean intensity fluorescence (MIF) was analyzed by FACS. Bar graphs represent the mean ± SEM of 5 independent experiments. * p < 0.05, ** p < 0.01.

Finally, we incubated neutrophils with the NADPH oxidase inhibitor diphenyleneiodonium (DPI) (10 μM) for 30 min, followed by vehicle, oleic acid, linoleic acid or GW9508 for 5 min. DPI significantly reduced ROS production in bovine neutrophils stimulated with the FFAR1 agonists (Fig. 8), suggesting the participation of NADPH oxidase in this response.

## Discussion

Due to the significant increase in plasma of free LCFAs in cows during peripartum, identifying the exact receptors for these fatty acids is crucial for understanding the physiological processes related to innate immunity in bovines. In this study, we demonstrated that the sequence initially identified as the bovine FFAR1 receptor, which was recently named a transcription variant of FFAR3, corresponds to a receptor for LCFAs that is highly homologous to human FFAR1. Analysis of cDNA and predicted amino acid sequences revealed that this protein has high identity to human FFAR1 (84%) compared with human FFAR3 (31%). Therefore, based on sequence analysis, we identified the presence of a FFAR1-like receptor in bovine.

Next, we assessed whether this receptor could be activated by long or short chain fatty acids. One method that is widely used to assess FFAR1 receptor activation is the analysis of intracellular calcium levels in heterologous expression systems [11,25]. We observed that only LCFAs, but not the short chain fatty acid propionic acid, increased intracellular calcium in CHO-K1 cells expressing bovine FFAR1. Additionally, the synthetic agonist of FFAR1, GW9508, also increased intracellular calcium levels. Reports by Itoh and Briscoe demonstrated that LCFAs are ligands of FFAR1, and they can increase intracellular calcium levels in CHO cells expressing human FFAR1 [11,25]. In bovine cells, oleic and linoleic acids increased intracellular calcium in neutrophils [4,9] and mammary epithelial cells [15]. In contrast, short chain fatty acids are ligands of FFAR3 and FFAR2 [26], receptors that are expressed in bovine neutrophils [37]. Furthermore, the short chain fatty acid, propionic acid, increases intracellular calcium in bovine neutrophils [37]. To confirm the existence of FFAR1 in bovine, we conducted experiments using the synthetic antagonist of the FFAR1 receptor, GW1100. In CHO-K1/bFFAR1 cells and bovine neutrophils, 10 μM GW1100 reduced the intracellular calcium mobilization induced by oleic and linoleic acid and GW9508. GW1100 is a selective antagonist of the FFAR1 receptor that dose-dependently inhibited the FFAR1-mediated increase in calcium stimulated by GW9508 and linoleic acid (pIC50 values of 5.99+/-0.03 and 5.99+/-0.06, respectively) in HEK cells expressing FFAR1, but GW1100 did not alter the GPR120-mediated stimulation of intracellular calcium release produced by both stimulus in HEK cells expressing GPR120 [13].

The ability of U73122 to reduce FFAR1 ligand-stimulated intracellular calcium mobilization in CHO-K1/bFFAR1 cells and bovine neutrophils suggests that PLC actively participates in this response. Activation of the human FFAR1 receptor activates G_q_ protein in pancreatic cells [16] and G_i/o_ in breast cancer cells [17], which leads to the activation of PLC and the generation of inositol triphosphate and diacylglycerol, the latter of which can activate PKC [16].

After identifying the existence of the FFAR1 receptor in bovine neutrophils, we assessed the functional role of this receptor in neutrophils. The release of MMP-9 granules induced by ligands of FFAR1 was reduced by GW1100, indicating that the FFAR1 receptor participates in this process. According to the classical cascade of G-protein coupled receptor activation, we demonstrated the participation of PLC and PKC in the FFAR1-ligand-mediated release of MMP-9. PLC was first reported to participate in MMP-9 release in human neutrophils activated with the neuropeptide, pituitary adenylate cyclase-activating protein, which acts through a specific G-protein coupled receptor [38]. PLC inhibition suggests the participation of a Gα_q_ protein in MMP-9 release after FFAR1 receptor activation in bovine neutrophils; however, we cannot discard the participation of another G protein, such as Gα_i_ protein, because different PLC isoforms can also be directly activated by Gβγ subunits [33,39,40,41,42]. In addition, we previously reported a partial decrease in intracellular calcium mobilization induced by oleic acid in bovine neutrophils treated with pertussis toxin, suggesting a partial role for the Gα_i_ protein [4]. FFAR1 receptor-activated MMP-9 release was PKC-dependent. Activation of the PLC/PKC pathway after FFAR1 activation in HepG2 cells was previously reported [43]. In human neutrophils, PKC and intracellular calcium participate in IL-8-mediated MMP-9 release [34]. Moreover, PKC has been described to participate in the MMP-9 release induced by TNF-α [44] or the agonist of G-protein coupled receptor B(1)R in human neutrophils [45].

Our results revealed that the FFAR1-ligand-mediated increase in ROS production was dependent of FFAR1, PLC, PKC and NADPH oxidase. A role for FFAR1 in superoxide production was previously suggested in pancreatic islets. Other reports demonstrated that the activation of rat pancreatic islets with palmitic acid, another LCFA FFAR1 agonist, increased NADPH oxidase-mediated ROS production and up-regulated FFAR1 protein [46], and the superoxide production induced by palmitic acid is dependent on FFAR1 and NADPH oxidase [47]. The participation of PLC and PKC in superoxide production in neutrophils is well documented. PLC isoforms (PLCβ2 and 3) are required for GPCR-induced superoxide release [33]. Additionally, PKC is required for the assembly of NADPH oxidase and activation of the respiratory burst in neutrophils [48].

In conclusion, we confirmed the presence and identity of a FFAR1 receptor in bovine neutrophils that is activated by LCFAs, such as oleic and linoleic acid, but not by short chain fatty acids, such as propionic acid. Furthermore, PLC-PKC signaling controls the release of MMP-9 granules and ROS production after FFAR1 receptor activation in bovine neutrophils. The results of this study highlight the link between metabolism and innate immunity, showing the importance of FFAR1 receptor on bovine neutrophils activation, which could contribute to the risk developing infectious diseases at calving, by mechanisms which have been involved in tissue damage. However, because FFAR1 receptor not only bind oleic or linoleic acids [11,13], future studies are necessary to assess the contribution of other LCFAs ligands for FFAR1 in the pathogenesis of inflammatory diseases, in order to establish their useful as potential biomarkers of disease risk, or to promote the presence of fatty acids beneficial for innate immunity through dietary or pharmacological approaches. It is interesting consider the known anti-inflammatory effect of omega-3 fatty acids via GPR120 receptor, which are also ligands for FFAR1 receptor [11,49], and a recent study suggested a potential anti-inflammatory role of FFAR1 receptor in bone tissue [50], however this finding could be tissue-specific and should be studied in detail.
